# Cardiac Protection by Oral Sodium Thiosulfate in a Rat Model of L-NNA-Induced Heart Disease

**DOI:** 10.3389/fphar.2021.650968

**Published:** 2021-04-15

**Authors:** Isabel T. N. Nguyen, Lucas M. Wiggenhauser, Marian Bulthuis, Jan-Luuk Hillebrands, Martin Feelisch, Marianne C. Verhaar, Harry van Goor, Jaap A. Joles

**Affiliations:** ^1^ Department of Nephrology and Hypertension, University Medical Center Utrecht, Utrecht, Netherlands; ^2^ Department of Pathology and Medical Biology, University Medical Center Groningen and University of Groningen, Groningen, Netherlands; ^3^ Clinical and Experimental Sciences, Faculty of Medicine, Southampton General Hospital and Institute for Life Sciences, University of Southampton, Southampton, United Kingdom

**Keywords:** hydrogen sulfide, hypertension, ACE-inhibitor, cardiovascular disease, nitric oxide

## Abstract

Hypertension contributes to cardiac damage and remodeling. Despite the availability of renin-angiotensin system inhibitors and other antihypertensive therapies, some patients still develop heart failure. Novel therapeutic approaches are required that are effective and without major adverse effects. Sodium Thiosulfate (STS), a reversible oxidation product of hydrogen sulfide (H_2_S), is a promising pharmacological entity with vasodilator and anti-oxidant potential that is clinically approved for the treatment of calciphylaxis and cyanide poisoning. We hypothesized that Sodium Thiosulfate improves cardiac disease in an experimental hypertension model and sought to investigate its cardioprotective effects by direct comparison to the ACE-inhibitor lisinopril, alone and in combination, using a rat model of chronic nitric oxide (NO) deficiency. Systemic nitric oxide production was inhibited in Sprague Dawley rats by administering N-ω-nitro-l-arginine (L-NNA) with the food for three weeks, leading to progressive hypertension, cardiac dysfunction and remodeling. We observed that STS, orally administered *via* the drinking water, ameliorated L-NNA-induced heart disease. Treatment with STS for two weeks ameliorated hypertension and improved systolic function, left ventricular hypertrophy, cardiac fibrosis and oxidative stress, without causing metabolic acidosis as is sometimes observed following parenteral administration of this drug. STS and lisinopril had similar protective effects that were not additive when combined. Our findings indicate that oral intervention with a H_2_S donor such as STS has cardioprotective properties without noticeable side effects.

## Introduction

Cardiovascular diseases represent a major global health problem and are a leading cause of morbidity and mortality worldwide. Hypertension is one of the most important risk factors for the development of various cardiac ailments, such as coronary heart disease, left ventricular hypertrophy, valvular heart disease and heart failure (Kjeldsen, 2018). The hemodynamic stress imposed by hypertension can induce cardiac remodeling involving cardiomyocyte hypertrophy, interstitial inflammation, fibrosis and microvascular rarefaction (Drazner, 2011). These maladaptive changes can eventually lead to left ventricular dysfunction and heart failure. Inhibition of the renin-angiotensin system (RAS) has shown clear benefit in this setting by reversing cardiac remodeling. However, despite widespread therapeutic use of RAS inhibitors and other antihypertensive therapies, patients still develop heart failure (Koitabashi and Kass, 2011). This demonstrates the ongoing need for novel therapeutic approaches that are effective and without major adverse effects.

Hydrogen sulfide (H_2_S) is the third endogenous gaseous transmitter, in addition to nitric oxide (NO) and carbon monoxide (CO) (Wang, 2010), and has many biological properties, including anti-oxidative, anti-apoptotic, pro-angiogenic, vasodilating properties and endothelial NO synthase (eNOS) modulating activities. At physiological pH, most H_2_S exists in the form of the hydrosulfide anion (HS^−^). As a powerful nucleophile and reducing agent it has the potential to modulate a wide range of pathophysiological processes (Liu et al., 2012; Pan et al., 2014). H_2_S is endogenously produced from the metabolism of l-cysteine by cystathionine ß-synthase (CBS) and cystathionine γ-lyase (CSE) and from d-cysteine by 3-mercaptopyruvate sulfurtransferase (3-MST) (Fu et al., 2012). In the presence of oxygen, HS^−^ can undergo oxidation to form sulfoxide species such as sulfite, sulfate, thiosulfate, polythionates, persulfides and polysulfides (Powell et al., 2018). In human physiology, H_2_S can be regenerated from per/polysulfides, sulfite and thiosulfate, but not from sulfate (Olson, 2011; Perridon et al., 2016).

Lower levels of endogenous H_2_S in plasma and tissues have been observed in various cardiovascular disease states, including heart failure, myocardial ischemia and atherosclerosis (Li et al., 2016; Pan et al., 2017), and exogenous treatment with H_2_S donors has been shown to be cardioprotective in various experimental models of cardiac injury. Although most of these investigations focused on acute myocardial protection, there are some studies on the protective effects of H_2_S in experimental models of chronic heart failure. In experimental models, H_2_S is often delivered *via* inhalation or by parenteral administration of simple inorganic salts such as sodium hydrosulfide (NaHS) and sodium sulfide (Na_2_S). A potential alternative exists in the form of sodium thiosulfate (STS, Na_2_S_2_O_3_), an endogenous metabolite that is produced during mitochondrial oxidation of H_2_S. In humans, short-term therapeutic intravenous and oral use of high concentrations of STS has been proven safe for the treatment of calciphylaxis (Singh et al., 2011), providing an opportunity for utilization of H_2_S-related therapies in other clinical settings. Recently, the intravenous administration of STS up to a dosage of 15 g was found to be well-tolerated and safe in patients with acute coronary syndrome (de Koning et al., 2020). Vasodilating and antioxidant properties have been attributed to STS (Sen et al., 2008). These beneficial effects would seem to make STS an attractive therapeutic candidate to tackle the cardiovascular alterations associated with hypertension.

Recently, we demonstrated that both orally administered and intraperitoneal injected STS improved renal function and damage in rat models of hypertension (Snijder et al., 2014; Nguyen et al., 2020). However, to the best of our knowledge, the effects of STS supplementation, compared to RAS inhibition, on cardiac dysfunction have not yet been investigated. In the present study, we sought to investigate the cardioprotective effects of orally administered STS in comparison to the angiotensin-converting enzyme (ACE) inhibitor lisinopril, alone and in combination, in a rat model of chronic NO deficiency. N-ω-Nitro-l-Arginine (L-NNA) was used to induce hypertension in conscious rats, as previously performed at our laboratory (Verhagen et al., 1999). Chronic inhibition of all NOS isoforms including eNOS, by either L-NNA or its methylester l-NAME (N-ω-Nitro-l-Arginine methylester), leads to dose-dependent severe hypertension, cardiac dysfunction and remodeling (Verhagen et al., 1999; Kobayashi et al., 2000; Biwer et al., 2013; Jin et al., 2017). This rat model was chosen as it is a reliable and reproducible model that develops hypertension within a short period of time, allowing us to evaluate the use of STS in hypertensive heart disease. Pharmacological inhibition of endogenous NO production in this model has the added advantage of simplifying the experimental landscape by removing an additional layer of complexity otherwise arising due to the chemical interactions between H_2_S and NO (Cortese-Krott et al., 2015), which can considerably complicate the interpretation of experimental results. We hypothesized that STS improves cardiac function, hypertrophy and damage in L-NNA-induced heart disease.

## Materials and Methods

### Animals

Male Sprague-Dawley rats (300–350 g, Envigo, Venray, the Netherlands) were cohoused in a climate-controlled facility with a 12-h light-dark cycle. Rats had access to water and standard rat chow (CRM-E; Special Diet Services, Witham, Essex, United Kingdom) *ad libitum*. All procedures were approved by the Animal Ethics Committee of the University of Utrecht (CCD: AVD1150020171484) and were in accordance with the Dutch Codes of Practice for the Care and Use of Animals for Scientific Purposes.

### Study Design

Following transportation, rats were allowed one week of acclimatization before experimental uses. After a baseline period, global NOS activity was inhibited by administering L-NNA (40 mg/kg/day; N5501–25G, Sigma-Aldrich, Zwijndrecht, the Netherlands) with the food for three weeks. After one week of inhibition of NOS, animals were divided into four groups 1) L-NNA only (N = 8), 2) L-NNA with STS (2 g/kg/day; 217247–500G, Sigma-Aldrich) administered *via* the drinking water for two weeks (L-NNA + STS; N = 7), 3) L-NNA with lisinopril (1 mg/kg/day; PHR1143-1G, Sigma-Aldrich) mixed with the food (L-NNA + lisinopril; N = 5) and 4) L-NNA with both lisinopril in the food and STS in the drinking water (L-NNA + STS + lisinopril; N = 5). The dose of STS was previously determined in a pilot study (Nguyen et al., 2020). The dose of lisinopril chosen for this study is very low in comparison to most other studies with lisinopril in models of heart disease, in order to allow observation of additional effects of STS. Starting at baseline, systolic blood pressure (SBP) was measured *via* tail-cuff plethysmography, and body weight was monitored every three days. Echocardiography was performed at baseline and after three weeks of L-NNA treatment. Reported baseline values for SBP and echocardiographic variables belong to the rats from the L-NNA group without any treatment and were measured before L-NNA treatment was initiated. At the end of the experiments, blood gas analysis and morphological analysis of the heart were performed in all four groups.

### Echocardiographic Evaluation

Transthoracic echocardiography was performed with a digital ultrasound machine (Sonos 5500, Philips Research, Eindhoven, the Netherlands) and a 15 MHz linear array transducer (Hewlett Packard, Palo Alto, CA). Rats were anesthetized with isoflurane (3% for induction and 2–2.5% for maintenance) and placed in supine position on a heating pad. Two-dimensional B-mode cine loops were recorded in the parasternal long-axis and the midpapillary short-axis views. Left ventricular (LV) volume was calculated with the area-length method at end-diastole and end-systole in the parasternal long axis view (Bongartz et al., 2010). Cardiac output and ejection fraction were then calculated from these LV volumes. Systolic and diastolic wall thickness and cavity dimensions were recorded in M-mode in the short-axis view. Images from apical 4-chamber view were acquired to evaluate LV filling and diastolic function. Mitral flow velocity tracings were obtained with pulsed-wave Doppler. Peak early E (E wave) and A (A-wave) filling velocities were measured. Tissue Doppler imaging as used to obtain early (e’) diastolic velocity at the medial mitral annulus. For evaluation of diastolic function, the ratio of peak velocity early (E) over late (A) mitral inflow and the ratio E over e’ was calculated. The recordings were coded for blinded analysis and analyzed offline using the system’s standard operating software. Variables were measured in at least three heart beats at end-diastole and end-systole.

### Blood Gas Analysis

Blood gas variables from the femoral artery were analyzed on a hand-held blood gas analyzer (i-STAT, Abbot, Hoofddorp, the Netherlands).

### Urine Collection for Oxidative Stress Measurement

For collection of 24 h-urines, rats were individually housed in metabolic cages without food but with access to drinking water supplemented with 2% glucose. Urinary excretion of lipid peroxides was measured as thiobarbituric acid reactive substances (TBARS) using a commercial kit (TBARS assay CAYM10009055–96, Cayman Chemical, Ann Arbor, MI) according to manufacturer’s instructions.

### Cardiac Histology

To investigate cardiac fibrosis, Masson’s Trichrome staining was used on paraffin-embedded cardiac sections. Subsequently, cardiac sections were scanned using a Hamamatsu Nanozoomer HT 2.0 (Hamamatsu Photonics, Japan). The extent of collagen deposition was determined using the Aperio Image Scope positive pixel count analysis V9.1 algorithm. The ratio between the fibrotic surface area and the total cardiac surface area was calculated. Histopathological analysis was performed in a blinded fashion.

To investigate cardiomyocyte cross-sectional area, formalin-fixed paraffin-embedded (FFPE) cardiac sections were deparaffinized, rehydrated and antigen retrieval in boiling Citrate buffer (10 mM, pH 6.0) was performed. Cardiac sections were incubated with Wheat-Germ Agglutinin (WGA)-FITC (1:200, L4895–2 mg, Sigma) conjugate for 60 min at room temperature. Nuclei were counterstained with DAPI (4,6-diamino-2-phenylindole dilactate (Invitrogen, Oregon United States) 1:40.000 in Fluoromount (DAKO Glostrup, Denmark). Stitched high-magnification (40x) scans were captured with TissueFAXS acquisition software on a Zeiss AxioObserver Z1 fluorescence microscope (TissueGnostics, Vienna, Austria). Subendocardial areas with cardiomyocytes in cross-sectional orientation were selected for analysis in ImageJ (version 1.53c; National Institutes of Health, Bethesda, MD). Cell boundaries were identified using Contrast Limited Adaptive Histogram Equalization (CLAHE), thresholding and binary options and cross-sectional cardiomyocyte area was measured in at least 1500 cells per sample.

### Statistical Analysis

Data were analyzed using GraphPad 7.0 Software (GraphPad, San Diego, CA). Data are presented as mean ± standard error of the mean (SEM). For longitudinal measurements, a two-way ANOVA for repeated measures was performed, followed by Bonferroni post-hoc test. Baseline values were only compared to the L-NNA group using a paired *t*-test and comparisons between the four groups were done using a one-way ANOVA, followed by Dunnett’s multiple comparison test.

## Results

### STS Treatment Decreases Systolic Blood Pressure

**FIGURE 1:**
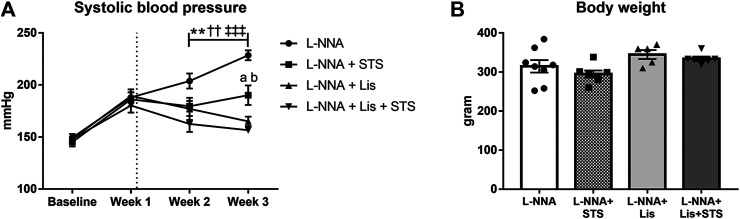
STS administration decreases systolic blood pressure. **(A)** Longitudinal measurements of systolic blood pressure and **(B)** body weight at the end of the experimental protocol are shown for the four groups. N = 5–8. *L-NNA vs. L-NNA + STS, ^†^L-NNA vs. L-NNA + Lis, ^‡^L-NNA vs. L-NNA + Lis + STS, ^a^L-NNA + Lis vs. L-NNA + STS, ^b^L-NNA + STS vs. L-NNA + Lis + STS. Two symbols *p* < 0.01, three symbols *p* < 0.001. Data previously published (Nguyen et al., 2020).

Chronic systemic NO deficiency was induced by administering L-NNA with the food (40 mg/kg/day) for three weeks (Nguyen et al., 2020). Administration for two weeks of either STS (2 g/kg/day) *via* the drinking water, the ACE-inhibitor lisinopril (1 mg/kg/day) *via* the food or their combination was started one week after initiation of NOS inhibition. As we previously reported (Nguyen et al., 2020), the administration of L-NNA rapidly induced progressive hypertension (Figure 1A) and treatment with STS was able to lower SBP measured by tail-cuff plethysmography. Treatment with lisinopril showed an antihypertensive effect, although at this low dose, SBP did not return to baseline levels. STS plus lisinopril did not further improve SBP compared to lisinopril alone. Body weights (BW) did not differ significantly between groups (Figure 1B).

### Oral Administration of STS Improves Systolic Function

**FIGURE 2:**
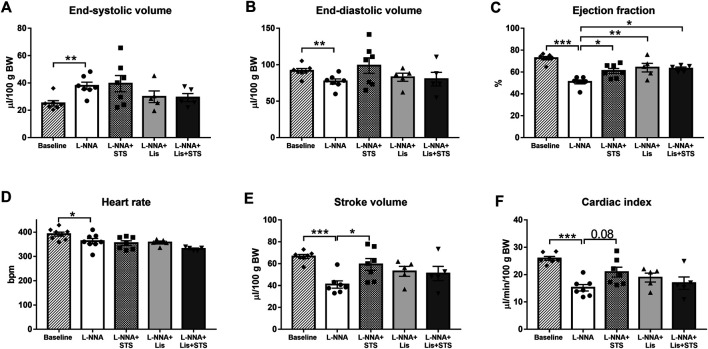
Treatment with STS improves systolic function. **(A)** End-systolic volume and **(B)** end-diastolic volume were measured to calculate **(C)** ejection fraction. **(D)** Heart rate and **(E)** stroke volume were used to calculate **(F)** cardiac index in the four groups. N = 5–8. *p < 0.05, **p < 0.01, ***p < 0.001.

Echocardiography was performed at baseline, before L-NNA administration was started, and after three weeks of L-NNA administration and two weeks of STS and/or lisinopril treatment. Chronic global NOS inhibition caused an increase in end-systolic volume (37.9 ± 2.6 vs. 25.1 ± 2.0 μL/100 g BW; Figure 2A) and a decrease in end-diastolic volume (77.0 ± 3.6 vs. 91.7 ± 3.1 μL/100 g BW; Figure 2B) and ejection fraction (72.2 ± 1.5% vs. 51.0 ± 1.7%; Figure 2C) compared to baseline values. Treatment with STS improved ejection fraction compared to L-NNA only (60.9 ± 2.3%). Lisinopril alone as well as the combination of STS and lisinopril also improved ejection fraction, however the combination therapy did not cause further improvement compared to either lisinopril or STS alone. Administration of L-NNA further caused a decline in heart rate (362 ± 11 bpm vs. 391 ± 8 bpm), stroke volume (41.0 ± 3.3 vs. 66.6 ± 1.9 μL/100 g BW) and cardiac index (15.2 ± 1.2 vs. 25.9 ± 0.7 μL/min/100 g BW) compared to baseline levels (Figure 2D–F). Oral administration of STS increased stroke volume (59.3 ± 5.4 μL/100 g BW) and tended to improve cardiac index (20.9 ± 1.8 μL/min/100 g BW; *p* = 0.08). As compared to STS, no differences were observed upon treatment with lisinopril or the combination therapy.

### L-NNA Administration did Not Affect Diastolic Function

**FIGURE 3:**
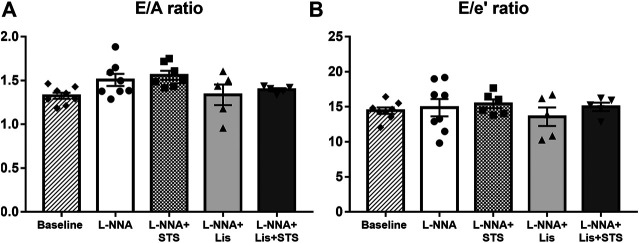
Diastolic function was not affected in L-NNA-induced hypertensive rats. Pulsed-wave and Tissue Doppler imaging were used to calculate **(A)** E/A ratio and **(B)** E/e’ ratio to evaluate diastolic function. N = 5–8.

Pulsed-wave Doppler and Tissue Doppler echocardiography were used to evaluate diastolic function. Chronic NO deficiency did not affect E/A ratio (Figure 3A) and E/e’ ratio (Figure 3B). Treatment with STS, lisinopril or the combination of STS and lisinopril also did not affect these variables.

### STS Administration Improved Left Ventricular Posterior and Septal Wall Hypertrophy

**FIGURE 4:**
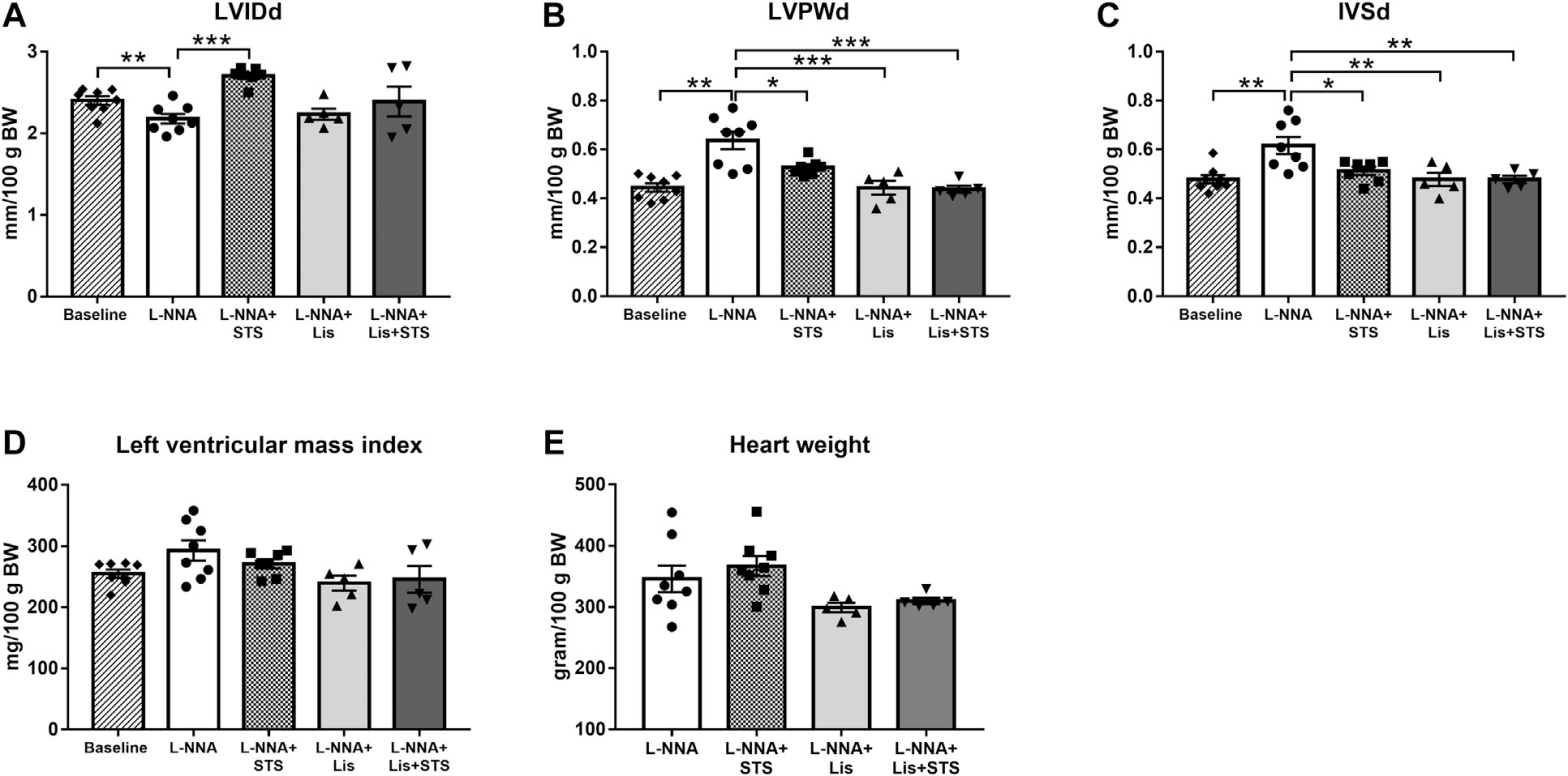
Treatment with STS improved left ventricular posterior and septal wall hypertrophy. **(A)** Left ventricular internal diameter (LVIDd), **(B)** left ventricular posterior wall thickness (LVPWd) and **(C)** interventricular septal wall thickness (IVSd) during diastole were measured *via* echocardiography. **(D)** Left ventricular mass index was calculated from these variables and **(E)** heart weight was measured at the end of the protocol. N = 5–8. *p < 0.05, **p < 0.01, ***p < 0.001.

M-mode echocardiography in the short axis at the level of the papillary muscles was performed in order to assess cardiac wall dimensions. Left ventricular internal diameter during diastole (LVIDd) was lowered after three weeks of L-NNA administration (2.2 ± 0.06 vs. 2.4 ± 0.05 mm/100 g BW) (Figure 4A). L-NNA further increased the thickness of the left ventricular posterior wall (LVPWd; 0.64 ± 0.04 vs. 0.45 ± 0.02 mm/100 g BW) and intraventricular septal wall during diastole (IVSd; 0.62 ± 0.04 vs. 0.48 ± 0.02 mm/100 g BW) (Figures 4B,C), indicative of left ventricular wall hypertrophy. Treatment with STS for two weeks increased LVIDd (2.7 ± 0.04 mm/100 g BW) and lowered LVPWd and IVSd (0.53 ± 0.01 and 0.51 ± 0.02 mm/100 g BW, respectively). Lisinopril also reduced LVPWd and IVSd thickness (0.44 ± 0.03 and 0.48 ± 0.03 mm/100 g BW) similar to the combination therapy (0.44 ± 0.01 and 0.48 ± 0.02 mm/100 g BW). Calculated left ventricular mass index and measured wet heart weight after three weeks of L-NNA administration was not different between groups (Figures 4D,E).

### Oral Administration of STS for Two Weeks Does Not Lead to Metabolic Acidosis

**FIGURE 5:**
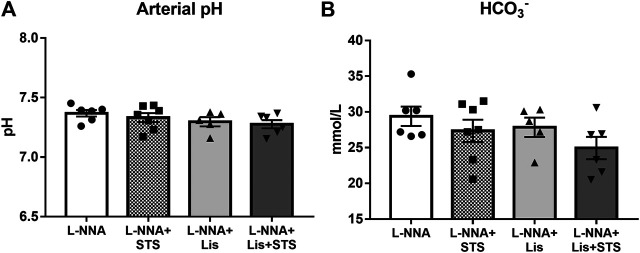
Oral treatment of STS does not lead to metabolic acidosis. **(A)** pH and **(B)** HCO_3_^−^ were measured in arterial blood *via* blood gas analysis at the end of the experimental protocol. N = 5–7.

After three weeks of chronic inhibition of NOS, blood gas analysis was performed on arterial blood samples to determine arterial pH (Figure 5A) and bicarbonate (Figure 5B) to assess the effect of STS on these variables. No differences were observed in arterial pH and bicarbonate between treatment groups, with or without STS.

### STS Treatment Reduced Oxidative Stress

**FIGURE 6:**
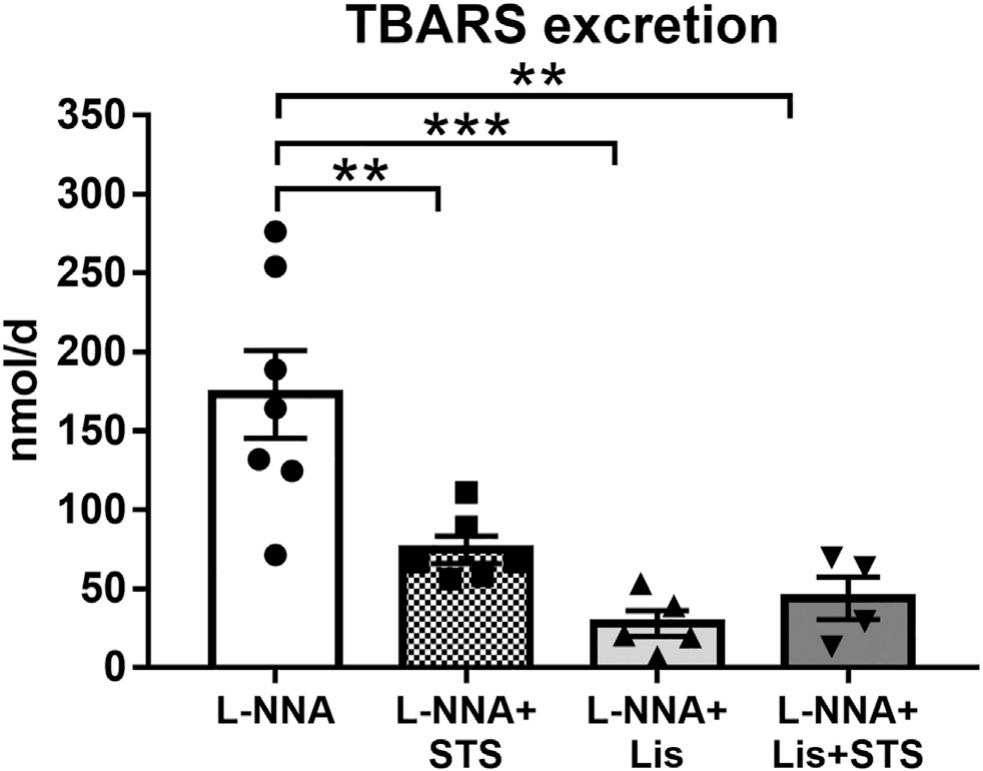
Oral treatment with STS reduces oxidative stress. The excretion of thiobarbituric acid reactive substances (TBARS) in urine was measured as a marker for systemic oxidative stress. N = 4–7. **p < 0.01, ***p < 0.001.

Systemic oxidative stress, as measured by the urinary excretion of TBARS, was lowered after treatment with STS (74.7 ± 8.7 vs. 173.1 ± 27.6 nmol/day) (Figure 6). Lisinopril also reduced TBARS excretion, as did the combination therapy (27.9 ± 8.5 and 43.9 ± 13.5 nmol/day).

### STS Administration Improved Cardiac Remodeling

**FIGURE 7:**
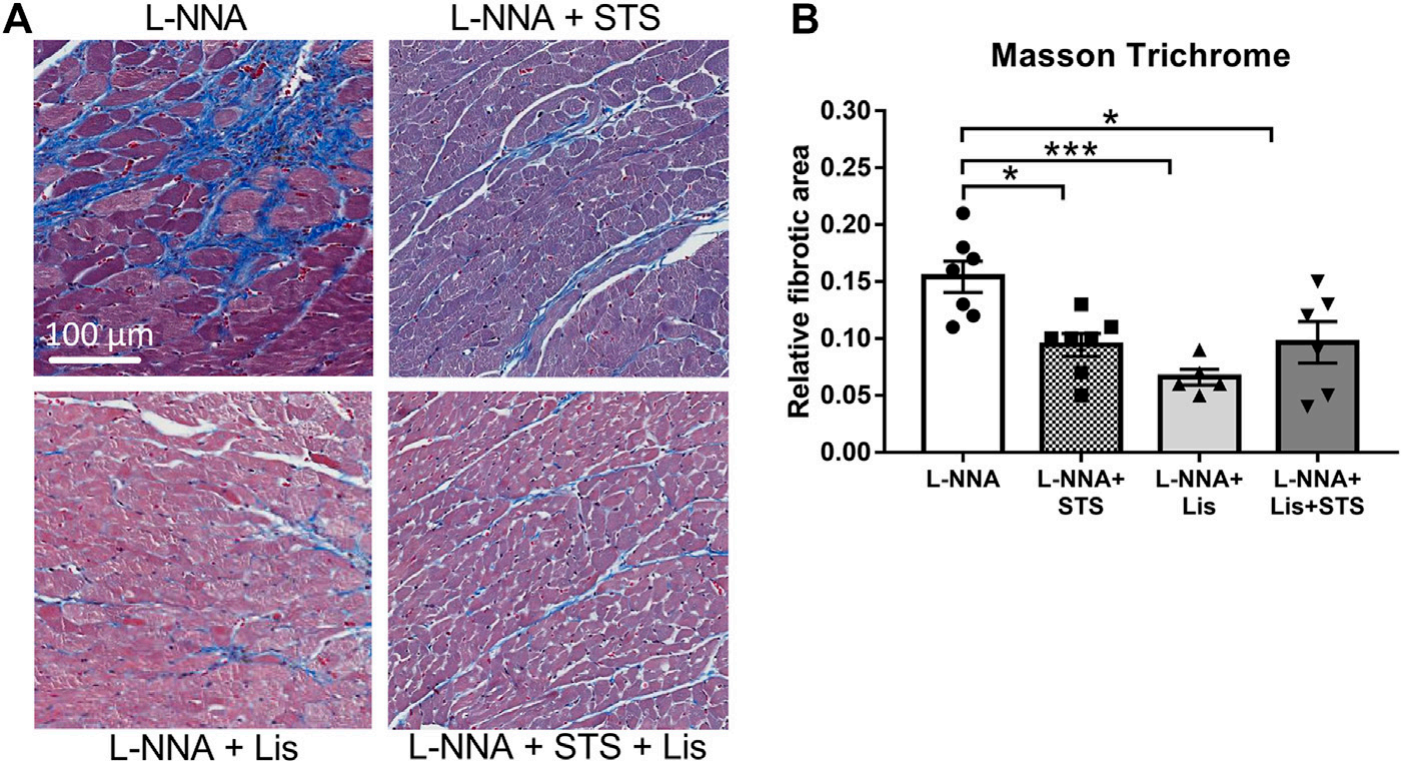
Treatment with STS reduced cardiac fibrosis. Masson Trichrome stained cardiac sections were used to visualize collagen deposition. **(A)** Representative images and **(B)** quantification of the fibrotic area in the four groups. N = 5–7. *p < 0.05, ***p < 0.001.

**FIGURE 8:**
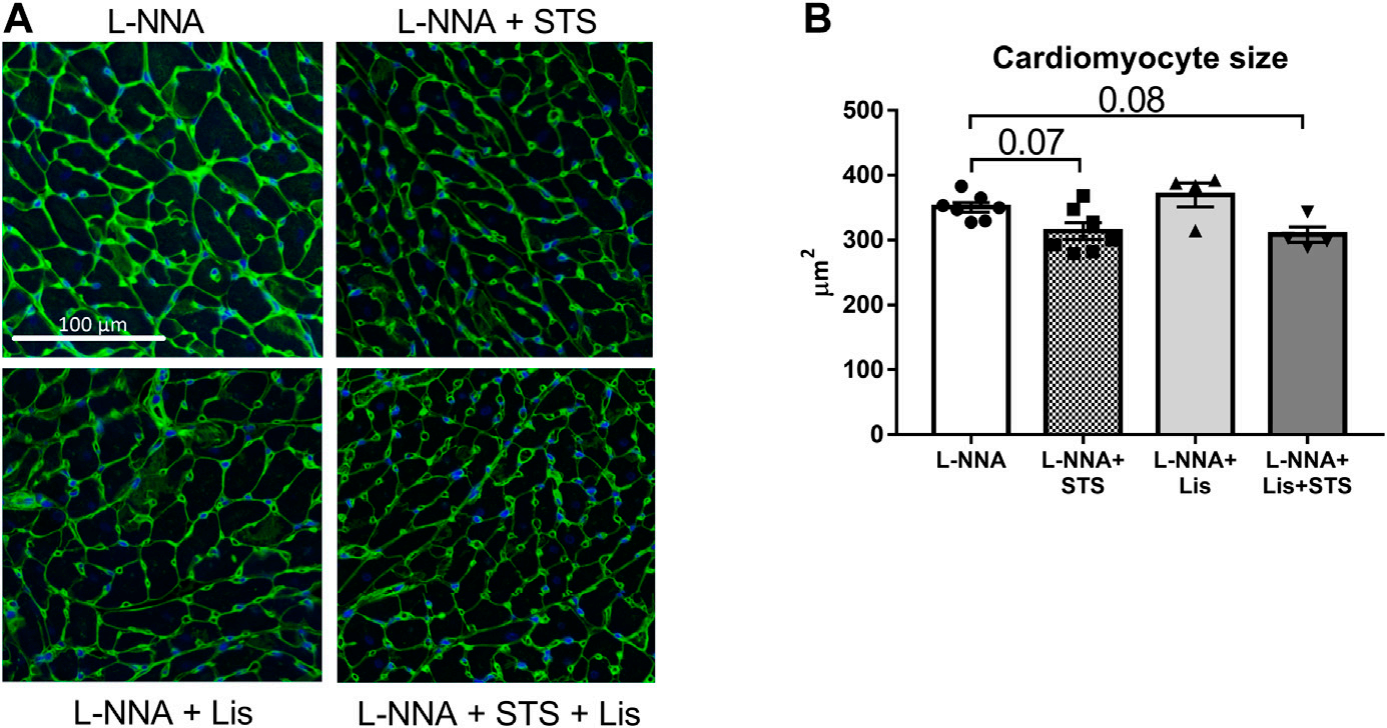
Treatment with STS tends to lower cardiomyocyte hypertrophy. Cardiac sections were stained with Wheat-Germ Agglutinin-FITC to investigate cardiomyocyte size. **(A)** Representative images and **(B)** quantification of the cardiomyocyte cross-sectional area in the four groups. N = 4–7.

Cardiac sections were stained with Masson Trichrome to determine collagen deposition following three weeks of L-NNA administration and two weeks of intervention (Figure 7A). A reduction in fibrosis was found after treatment with STS or lisinopril (0.094 ± 0.01 and 0.066 ± 0.01 vs. 0.15 ± 0.01%) compared to L-NNA alone (Figure 7B). Combination of STS with lisinopril did not further improve fibrosis compared to STS or lisinopril alone. Cardiac sections were stained with Wheat-Germ Agglutinin -FITC to assess cardiomyocyte cross-sectional area (Figure 8A). There was a trend toward a lower cardiomyocyte size in the STS- and STS + Lisinopril-treated rats (*p* = 0.07 and *p* = 0.08, respectively) compared to L-NNA only (Figure 8B). At this low dose of lisinopril, no difference was observed compared to the L-NNA group.

## Discussion

The major finding of our study is that oral administration of STS *via* the drinking water ameliorates L-NNA-induced heart disease to the same extent as ACE inhibition. Treatment with STS for two weeks improved systolic function, left ventricular wall hypertrophy, cardiac fibrosis and systemic oxidative stress. No signs of metabolic acidosis were observed following oral administration of STS. This indicates that oral intervention with the H_2_S donor STS is safe and has promising cardioprotective effects.

### Oral STS Attenuated L-NNA-Induced Hypertension

The reduction in blood pressure by orally administered STS observed in this study (Nguyen et al., 2020) is in line with previous findings with parenteral treatment with NaHS. Early studies on H_2_S delivery showed that the use of aqueous NaHS in rat aortic rings induced marked relaxation revealing the vasorelaxant properties of H_2_S (Zhao et al., 2001). Furthermore, administration of NaHS lowered blood pressure in experimental models of hypertension (Zhong et al., 2003; Yan et al., 2004; Ahmad et al., 2014; Snijder et al., 2014; Xiao et al., 2016). We observed a similar blood pressure reduction after lisinopril administration and no additive effect with the combination therapy. It is well-known that inhibition of the RAS system is able to normalize arterial blood pressure in hypertensive heart disease both in spontaneously hypertensive rats (Brilla et al., 1996) and in rats with L-NNA-induced hypertension (Verhagen et al., 2000). Additionally, ACE-inhibitors inhibit the degradation of bradykinin, thereby increasing their blood pressure lowering effects (Grafe et al., 1993). The mechanism of blood pressure reduction observed with STS in our study remains unknown. STS may react *via* various thiol reactions involving transsulfuration enzymes with cysteine to produce H_2_S in the smooth muscle cells (Sowers and Hayden, 2010). It has been shown that H_2_S can affect the RAS system directly, by inhibiting the activity of renin and ACE, by interfering with zinc at the active site of ACE (Laggner et al., 2007; Lu et al., 2010). This suggests that STS and lisinopril might act *via* the same pathway to lower the blood pressure, which would also explain why the antihypertensive effects were not additive.

Other proposed mechanisms for the vasodilating effects of H_2_S include the activation of K_ATP_ sensitive channels through cysteine-S-sulfhydration, induction of Keap1/Nrf2 signaling and augmentation of endogenous NO signaling (Jiang et al., 2010; Mustafa et al., 2011; Huang et al., 2016; Nabeebaccus and Shah, 2018). H_2_S also prevents the inactivation of cyclic adenosine monophosphate and cyclic guanosine monophosphate by nonselective inhibition of phosphodiesterases (Bucci et al., 2010). Furthermore, STS may recouple endothelial NOS *via* restoration of tetrahydrobiopterin to its fully reduced state, thereby restoring bioavailable NO (Whiteman and Moore, 2009). However, since our model relies on the systemic inhibition of endogenous NOS production, we believe that these mechanisms cannot explain the effects observed in the current study.

In addition, oxidative stress can contribute to the development of hypertension by reducing endothelial NO bioavailability (Schulz et al., 2011). Both STS and lisinopril treatment had anti-oxidant effects, evidenced by lower TBARS excretion. Our findings suggest that the antihypertensive effect exerted *via* STS or lisinopril may be mediated *via* common pathways associated with oxidative stress and that treatment with STS might be beneficial for patients with hypertensive heart disease that respond poorly to RAS inhibition. However, for the same reasons as alluded to above, this mechanism is unlikely to be mediated *via* an enhancement of endogenous NO availability. It is possible that the observed reductions of lipid oxidation products in urine were the result of acute anti-inflammatory effects or due to a modulation of cellular mitochondrial function; dedicated experiments would be required to confirm or exclude these possibilities, which was beyond the purpose of the current study.

### Oral STS Improves Systolic Function and Cardiac Remodeling

Growing evidence shows that H_2_S improves cardiac function and remodeling in different cardiovascular disorders, such as myocardial ischemia reperfusion injury, myocardial infarction, cardiac arrhythmia and heart failure (Shen et al., 2015). Treatment with NaHS attenuated cardiac dysfunction, evidenced by increased ejection fraction, and reduced cardiac fibrosis in rats with myocardial infarction and heart failure (Pan et al., 2014; Abdelmonem et al., 2019). Treatment with STS for six weeks prevented the development of ventricular contractile dysfunction, thickening of the left ventricular posterior wall and collagen deposition in mice with chronic heart failure (Sen et al., 2008). Intraperitoneal injections with STS reduced the degree of cardiac hypertrophy and fibrosis in angiotensin II-induced hypertensive rats (Snijder et al., 2015). STS might trigger the regulation of matrix metalloproteinases in the failing heart, thereby attenuating collagen deposition in the extracellular matrix (Sen et al., 2008).

In our study, treatment with STS was started one week after L-NNA-induced hypertension was established, whereas in previous studies STS treatment is often initiated together with disease induction. Here we observed that orally administered STS improved systolic function and reduced cardiac fibrosis. The low dose of the ACE-inhibitor lisinopril, purposely chosen to allow observation of additional effects of STS, improved cardiac function and remodeling similar to treatment with STS. Additionally, intervention with STS on top of lisinopril did not perform better compared to either STS or lisinopril alone. Lisinopril has been shown to enhance collagen degradation by activation of tissue matrix metalloproteinase 1 in spontaneously hypertensive rats (Brilla et al., 1996). As for hypertension, this suggests that the antifibrotic response exerted *via* STS or lisinopril is mediated *via* common pathways.

It has been reported that NaHS treatment ameliorated l-NAME-induced cardiac dysfunction and remodeling *via* activation of the Akt/eNOS/NO pathway mediated by K_ATP_ channels (Jin et al., 2017). H_2_S markedly prevented the development of cardiac fibrosis and decreased the collagen content in the cardiac tissue by inhibiting the activity of intracardiac angiotensin II (Huang et al., 2012). In addition, the suppression of inflammation, the reduction of cardiac apoptosis from oxidative stress and improving mitochondrial derangements are likely to have antifibrotic roles under hypertensive conditions (Wang et al., 2011; Kang et al., 2020). Antihypertensive effects of H_2_S, *via* modulation of channel activities and the cGMP/PKG pathways, may also contribute to the suppression of fibrosis caused by hypertension (Meng et al., 2015).

Chronic NO inhibition by l-NAME has been shown to induce cardiomyocyte hypertrophy in rats (Sanada et al., 2001). In our study orally administered STS tended to decrease cardiomyocyte cross-sectional area. Parenteral treatment with STS or NaHS attenuated cardiomyocyte hypertrophy in angiotensin II induced hypertensive rats (Snijder et al., 2015). Although ACE-inhibition effectively reduces cardiomyocyte size (Tamura et al., 2000), the low dosage of lisinopril used in this study did not decrease cardiomyocyte cross-sectional area. Our findings suggest that oral intervention with STS might be beneficial in cardiovascular disorders associated with a reduced left ventricular contractile function and cardiac remodeling.

### Metabolic Acidosis

For the administration of H_2_S in experimental models, soluble sulfide salts are often used while occasionally gaseous H_2_S has been administered. In clinical practice, STS has been approved in two indications: 1) together with sodium nitrite, as antidote for cyanide intoxications, and 2) for the short-term treatment of calcific uremic arteriolopathy in patients with end-stage kidney disease. In both indications, intravenous infusion of STS is the preferred route of administration. In some cases, such patients developed metabolic acidosis with an anion gap following parenteral infusion with STS (Rein et al., 2014; Hunt and Ryder, 2018). In our study we showed that STS, when orally administered *via* the drinking water, did not lead to metabolic acidosis, suggesting that oral treatment with STS might be a more advantageous route of administration. It should be noted that kidney function is only reduced by about 50% in this model (Verhagen et al., 1999). We cannot exclude that even oral STS may lead to metabolic acidosis when kidney function is more severely compromised.

### Oral Treatment With STS Reduces Oxidative Stress

Previously we have shown that various conditions associated with systemic oxidative stress are accompanied by increased urinary TBARS excretion, namely chronic kidney disease induced by subtotal nephrectomy (Papazova et al., 2014), chronic hypertension in spontaneously hypertensive rats (Racasan et al., 2004; Koeners et al., 2011), and dietary hypercholesterolemia (Attia et al., 2003). Importantly, cross-transplantation of healthy and diseased kidneys in healthy recipients or rats with long-standing chronic kidney disease convincingly showed that urinary TBARS excretion in these animals represents systemic but not intrarenal oxidative stress (Papazova et al., 2018). The production of reactive oxygen species causes endothelial dysfunction and can therefore also contribute to the development of hypertension (Schulz et al., 2011). H_2_S is a known antioxidant by increasing the levels of glutathione and directly scavenging reactive oxygen species (Kimura et al., 2010). In addition to its vasodilator action, eNOS-derived NO is also a potent antioxidant with cardioprotective effects (Erkens et al., 2018), and we cannot exclude that L-NNA induced global NOS inhibition in our model may have sensitized the heart to other antioxidants effects. Parenteral treatment with STS has been shown to reduce the levels of oxidative stress in angiotensin II-induced hypertensive rats (Snijder et al., 2015). STS might act as an antioxidant by scavenging superoxide in the myocardial tissue, thereby helping to rescue the failing heart (Sen et al., 2008). Here we showed that oral treatment with STS reduces systemic oxidative stress as evidenced by a lower TBARS excretion. These antioxidant effects of STS, in addition to its vasodilating properties, may have also contributed to the cardioprotection observed in this study.

## Conclusion

Orally administered STS improves systolic function, and ameliorates hypertension, left ventricular hypertrophy, fibrosis and systemic oxidative stress, all to the same extent as ACE inhibition. These findings indicate that oral treatment with STS has marked cardioprotective properties and shows therapeutic potential in hypertensive heart disease.

## Data Availability

The raw data supporting the conclusions of this article will be made available by the authors, without undue reservation.
